# Therapeutic Strategies Targeting Cancer Stem Cells and Their Microenvironment

**DOI:** 10.3389/fonc.2019.01104

**Published:** 2019-10-24

**Authors:** Hao-Ran Sun, Shun Wang, Shi-Can Yan, Yu Zhang, Peter J. Nelson, Hu-Liang Jia, Lun-Xiu Qin, Qiong-Zhu Dong

**Affiliations:** ^1^Department of General Surgery, Cancer Metastasis Institute, Institutes of Biomedical Sciences, Huashan Hospital, Fudan University, Shanghai, China; ^2^Medizinische Klinik und Poliklinik IV, Ludwig-Maximilian-University (LMU), Munich, Germany

**Keywords:** cancer stem cells, tumor microenvironments, therapeutic resistance, mechanism, strategy

## Abstract

Cancer stem cells (CSCs) have been demonstrated in a variety of tumors and are thought to act as a clonogenic core for the genesis of new tumor growth. This small subpopulation of cancer cells has been proposed to help drive tumorigenesis, metastasis, recurrence and conventional therapy resistance. CSCs show self-renewal and flexible clonogenic properties and help define specific tumor microenvironments (TME). The interaction between CSCs and TME is thought to function as a dynamic support system that fosters the generation and maintenance of CSCs. Investigation of the interaction between CSCs and the TME is shedding light on the biologic mechanisms underlying the process of tumor malignancy, metastasis, and therapy resistance. We summarize recent advances in CSC biology and their environment, and discuss the challenges and future strategies for targeting this biology as a new therapeutic approach.

## Introduction

Cancer remains one of the leading causes of death worldwide (1). Tumor malignancy is linked to tumor heterogeneity, which has been proposed to be driven by a minor subpopulation of cancer cells referred to as cancer stem cells (CSCs) (2, 3). This subpopulation of tumor cells have the capacity to sustain tumorigenesis and drive tumor heterogeneity, processes that underlie tumor progression, metastasis, and resistance to anti-cancer therapies (4). To date, CSCs identification has been largely based on surface markers as well as their ability to self-renew and propagate. However, CSC surface markers alone are not a reliable means of identifying these populations which has led to some confusion and controversy in the field. It is unlikely that these methods can afford a universal specific marker for the identification of these cells. However, some functional markers including the ATP-binding cassette (ABC) transporter and aldehyde dehydrogenase (ALDH) activity (5), the activation of some key signaling pathways (6), live-cell RNA, and single-cell DNA detection (7) have been found to improve CSCs identification in some instances.

The self-renewal potential and extensive clonogenic properties of CSCs are dependent on the tumor microenvironment (TME) (8, 9). The interaction between CSCs and their tumor niche is strongly linked to the characterization of CSCs (10). Through this interaction, CSCs are able to preserve the tumor heterogeneity that underlies the important malignant behaviors of invasion, metastasis, and therapy resistance (11). The influence of TME on CSCs physiology has been shown to act through intrinsic and extrinsic actions. The intrinsic mechanisms include DNA methylation or demethylation, and gene mutation, while the extrinsic actions involve the production of diverse growth factors and cytokines by the TME leading to the activation of specific signaling pathways (12). In addition, many studies have shown that CSCs may be responsible for tumor resistance to conventional cancer therapy (4, 13). The resistance that is enhanced through the cross-talk between CSCs and the TME include activation of the DNA repair system (8, 14), increased resistance to hypoxic environments (15), and the phenomenon of epithelial to mesenchymal transition (EMT) (16). These features may help explain the therapeutic failures that are often encountered in different tumor settings.

Despite the enormous challenges seen, a series of promising new therapeutic approaches based on this biology are currently under development. Notably, targeted therapeutic approaches have emerged as important tools in treatment strategies. To this end, both the CSCs and the TME represent important therapeutic targets. Emerging research has shown that CSC-targeted approaches have proven to be effective in prolonging survival time (17). In this review, recent advances in CSC biology are summarized, and the potential challenges and future strategies for targeted therapy and combination therapy to eliminate both cancer and CSC populations are discussed.

## Identification of CSC

CSCs are a small subpopulation of cells with characteristics that include the capacity to cycle slowly, self-renew, and initiate a novel tumor (18–20). Leukemic stem cells (LSCs) were first described in acute myeloid leukemia (AML), where it was demonstrated that CD34^+^CD38^−^ AML include a subpopulation of LSCs with a capacity to differentiate and self-renew (20). The early study first demonstrated the existence of a unique tumor subpopulation with the ability to drive tumor progression and recurrence. CSC-like subpopulations have been subsequently isolated from a variety of solid tumors (21). Because some CSCs have been identified via specific surface markers, a door has been opened for potential targeted therapy approaches directed against these cells (22). However, because of the wide diversity underlying this general biology across tumor types, general markers for the global identification of these cells are not available.

The functional relevance of surface markers for CSC identification is still disputed (23, 24). It is suggested that CSCs may arise from normal stem cells, progenitor cells, or even more differentiated cells. In tumor patients, the expression of CSC surface markers in normal organs implies potential metastasis and poor prognosis (25). CD24, CD26, CD44, CD133, CD166, and EpCAM (epithelial cell adhesion molecule, CD326) are surface markers commonly used in CSC characterization (26). CD133 is a special marker that has been widely used for identifying CSCs in different tumor settings (27), especially in solid tumors, such as prostate (28), pancreas (29), brain (30), liver (31), colorectal (32), ovarian (33), osteosarcoma (34), and lung cancer (35). In colorectal cancer, a subpopulation of cells expressing CD133, which comprise 1% of the tumor cells, was shown to efficiently induce xenografts *in vivo* (36). However, CD133 expression appears to represent only one subset of CSCs and the surface marker can also be found to be ubiquitously expressed on many differentiated cells (37). EpCAM is expessed by most adenocarcinomas and is thought to participatein tumor progression (38). In liver and pancreatic cancer (29, 39), a high expression of EpCAM is associated with the dedifferentiation of tumor cells that have regained stem cell-like features. CD24 is highly expressed in embryonic stem cells (40) and has been widely detected in different tumor settings. The combined surface markers C44/CD24 have been used to identify CSCs in breast tumors (41, 42). CD26 (dipeptidyl peptidase-4, DPP4) is expressed on various cell types, which includes cells with stem traits and is thought to influence progenitor cell migration (43). CD26 is widely detected in leukemic and colorectal cancer (44). Aldehyde dehydrogenase 1A1 (ALDH1A1) has also been identified as a potential CSC marker. ALDH expression is associated with the oxidation of aldehydes to carboxylic acid. ALDH activity has proven useful for the prediction of poor tumor outcome in prostate, breast and lung cancer (45, 46). The ABC transporters are able to pump chemotherapy agents out of the cells that express these proteins. These transporters are widely expressed by CSCs and are thus thought to represent an important component for the failure of cancer chemotherapy. The expression of ABC transporters has been used to identify or isolate CSCs from solid tumors (47). Importantly, CSCs have also been functionally identified in what would represent CSC negative populations based on surface markers (48). Thus, it is generally important to make use of multiple markers to more reliably identify CSCs. To this end, the activation of CSC-related signaling pathways such as the canonical Wnt pathway, has been shown to provide an addition level of information to better identify CSCs from colon and ovarian cancer (49).

Some surface markers used to characterize CSCs are also expressed by normal stem cells. CD29 (integrin β1) is widely expressed on CSCs and also on some normal cells, and is regarded as a marker for breast cancer CSCs. CD29 is important for breast cancer cell adhesion to extracellular matrix, and is thought to promote self-renewal and chemoresistance (50). CD9 (MRP-1) is widely expressed in normal tissues. However, it can also act as an effective marker to diagnose B-acute lymphoblastic leukemia (B-ALL) and is linked to drug resistance. CD44s is frequently used as a CSC marker (51). CD44 is composed of different subtypes (CD44V1-V10) (52, 53) and is expressed by both CSCs and normal cells. CD44 expression is associated with cancer progression and metastasis (51). For example, the CD44V9 is a predictive marker in solid tumors, including head and neck squamous carcinoma and gastric cancer. CD44V3 and V6 have been shown to be linked to invasion, metastasis, and resistance to apoptosis in colorectal cancer (54). The CD44V3-7 varients are highly expressed in non-small cell lung carcinoma (NSCLC) (55, 56). In addition, CD44V6 is associated with lymph node metastasis (6). In examples of breast cancer, high expression of CD44V3, V5, and V6 have been detected and shown to be related to the invasive properties of the tumor (57, 58). ABCB5 (ATP-binding cassette transporter) is a member of the ATP-binding cassette transporter family. ABCB5 expressed by normal cells and contributes to cell proliferation and differentiation (59). However, the expression of ABCB5 has also been demonstrated in several malignant stem cells, including ocular surface squamous neoplasm (OSSN) (60) and melanoma (61, 62). The ABCB5 subpopulation was shown to have an unlimited self-renewal potential, and is thought to foster tumor progression, metastasis, and therapy resistance (63, 64).

CSCs with unlimited self-renewal potential express potential specific markers that can help dinstinguish them from other cells. By making use of markers in CSCs, it may be possible selectively eradicate CSCs in various tumors (22, 65). While there is a growing list of markers that have been used for identification and isolation of CSCs, very few reliable specific surface markers have been found that clearly identify CSCs because CSCs, for the most part, are heterogeneous. The identification of more universal CSC markers across diverse cancer types would clearly redine the field. Finally, what is emerging is that the application of multiple markers used in combination represents the most reliable means of characterizing these cells absence the functional criteria used to define CSCs.

## CSC Microenvironment

Accumulating evidence suggests that cancer cells acquire a “stemness” feature in part through environment input. Because of this, even differentiated cancer cells can revert to a more dedifferentiated state which has been linked to the ability to form tumors (66). CSCs co-injected into mice with stromal cells extracted from a tumor environment form more aggressive tumors that do CSCs alone suggesting an important role for the stromal matrix surrounding CSCs, also known as the “CSC niche” (67, 68). Cancer cells in a such a niche are capable of maintaining their stemness state (12, 69). The niche can contain various cell types and growth factors providing a tumor promoting microenvironment. This can involve endothelial cells, immune cells, cancer associated fibroblasts (CAFs), various growth factors, and cytokines. In addition to these components, environment changes, such as hypoxia, and pH have been proposed to contribute to the CSC niche (70–72). The perivascular niche, which is best studied in brain tumors, is recognized as a hallmark of glioblastoma (GBM). The perivascular niche enhances GBM stemness and ability for self-renewal and invasion (73).

Low levels of oxygen, referred to as hypoxia, is an important feature of TME. Hypoxia appears to help drive the maintenance of stemness and thus malignancy of CSCs, which promotes tumor survival and metastasis (74). The hypoxia-inducible factors (HIFs) are transcription factors that are increased in response to t hypoxia, and high expression of HIFs (HIF-1α, HIF-2α) is correlated with tumor malignancy (75). The octamer-binding transcription factor 4 (Oct4) is activated by HIF-2α and is linked to control of CSCs self-renewal and an increase in the malignant potential of embryonic stem cell-derived tumors (76). Another transcription factor-Sox2 is also linked to stemness through modulation of Oct4 levels in CSCs (77). A reduction in miR-145 was shown to significantly reduce expression Oct4 and Sox2, and thus lead to a decrease in the CSCs population and chemosensitivity in colon cancer (78). In pancreatic ductal adenocarcinoma (PDAC), YAP/HIF-1α signaling is activated by HGF stimulation through its receptor cMET (79). Dysregulation of YAP is related to tumor proliferation, epithelial mesenchymal transition (EMT) and therapy resistance. In the context of a low oxygen environment, CSCs can obtain energy by both OXPHOS and glycolysis activity. During hypoxia, glycolytic enzymes and glucose transporters become induced by HIF-1. Pyruvate dehydrogenase kinase 1(PKD1) plays a role in converting pyruvate to acetyl-coenzyme A (80). As an essential glycolytic enzyme, PDK1 is associated with tumor proliferation, metastasis and poor prognosis (81). In breast cancer, PDK1 stimulates glycolytic activity to stimulate cancer cells to take on stemness traits (82). The use of PDK1 inhibitors can help block glycolysis activity and also limit maintenance of breast cancer stem cells (82). In many instances, CSCs have been shown to be primarily glycolytic, or to preferentially shift from OXPHOS to glycolysis in a tumor type-dependent manner. In lung cancer (83), glioblastoma (84), and PDCA (85), CSCs were also shown to utilize OXPHOS as the preferred energy production process, however, the mechanism is still unclear. To target this metabolic biology it is thought that a combined therapy targeting both aerobic glycolysis and OXPHOS dependent cells may be the most effective therapy to block CSCs.

CAFs, as a part of TME, are believed to drive tumor progression and dedifferentiation by their secretion of key growth factors and their interplay with other stromal cells. HGF secreted by CAFs was found to activate the canonical Wnt pathway and promote cancer cells to dedifferentiate to the CSCs state (26). Cytokines secreted by CAFs, such as CCL2, IGF-1, and TGF-β affect the expansion and self-renewal of CSCs in breast, lung and gastric cancer (86, 87). In a hypoxic environment, CD44 is highly expressed by CAFs that in turns helps mediate cancer cell migration and stemness sustainability (88). The high-mobility group box 1 (HMGB1) released from CAFs was demonstrated to stimulate CSCs through the TLR4 receptor in breast cancer (89). CAFs induced expression of Notch3 is responsible for the activation of lysine demethylase 1 (LSD1) in CSCs, driving self-renewal in HCC (90, 91). In addition, CAFs facilitate tumor cells migration and metastasis indirectly through EMT. In prostate cancer, CAFs secret CXCL12 and promote EMT by inducing the expression of CXCR4 (one of the EMT phenotypes), which enhances metastasis (92). Recent reports also show that CSCs can differentiate into CAF-like cells through TGF-β secretion that promotes self-renewal and proliferation (93). CSCs also secret the Hedgehog ligand SHH that is known to increase the proliferation of CAF in the mammary tumor, and the CAFs secret factors to improve the ability of CSCs malignancy (94).

**Figure 1:**
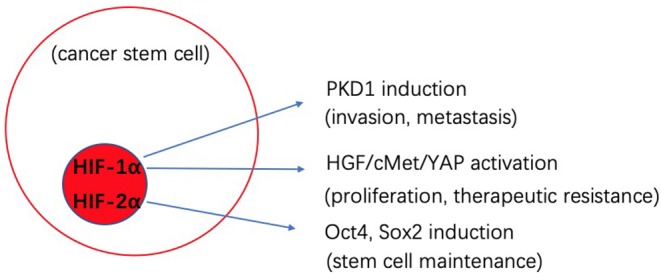
Schematic illustration of the potential effects of hypoxia interaction on cancer stem cells (CSCs).

**Figure 2:**
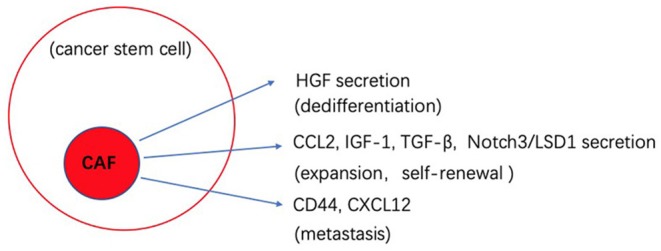
Schematic illustration of the potential effects of CAF interaction on cancer stem cells (CSCs).

The biological cross-talk between CSCs and TME is quite complicated, and changes between different tumors and environments. By better understanding these processes, we can develop novel strategies to better target CSCs. Figures 1, 2 gives insight into illustration of hypoxia and CAF interactions on CSC.

## Therapeutic Resistance Driven by CSC and their Microenvironment

Tumors recurrence often means poor prognosis and increased resistance to therapy (95, 96). Increasing evidence suggests that the interplay between CSCs and their TME is important in tumor development. The tumor is a complex tissue composed of different subpopulations of tumor cells and tumor-associated stromal cells. Through interaction with the microenvironment, CSCs can avoid target exposure and this may be a key to therapy resistance (97–99).

The general therapeutic resistance of CSCs allows them to escape from elimination and re-establish tumor. When the treatment cycle comes to an end, CSCs are revived from their quiescence and promote tumorgenesis (100, 101). The mechanisms by which CSCs achieve therapeutic resistance involves heightened DNA damage repair capacity, high expression of multiple drug resistance (MDR) transporters and high expression of anti-apoptosis proteins (102–105).

The DNA damaging repair (DDR) system is important in tumor progression. When under chemotherapy or radiotherapy, damaged DNA triggers the DDR, which enables CSCs to survive and thereby remain resistent to treatment. Several pathways can be activated in cancer cells includiong the double-strand breaks (DSBs) repair (homologous and non-homologous end joining), base excision repair (BER), transcription coupled nucleotide excision repair (NER), and mismatch repair (MMR) systems (106). Previous study have showen that the high expression of apurinic/apirimidinic endonuclease/redox effector factor (Ape1/Ref-1), corresponding to an activation of the BER pathway, has been implicated in the development of CSCs (107). Overexpressed Ape1/Ref-1 was also shown to maintain a low level of reactive oxygen radicals (ROS) that prevented DNA damage and cell death in CSCs (108–110). The Mre11-Rad50-Nbs1 (MRN) complex has the capacity to repair DNA and modulate cells apoptosis, and gene stability and is an important part in the DSBs pathway (111, 112). In nasopharyngeal and gastric cancer, MRN-ATM meditated DNA repair induced resistance to common chemotherapy agents, such as cisplatin and 5-FU (113, 114). The MRN complex also acts as one of the DSB pathway key elements to produce radio-resistance in various cancer types (115). Transcription factors such as forkhead box protein m1 (FOXM1), P53, glioma-associated oncogene (GLI1), and c-MYC, were also shown to be important for the DNA repair response (116, 117). Treating colon cancer with doxorubicin (a chemotherapy agent) was shown to lead to the activation of SMAD, which binds to P53 to produce chemoresistance (118).

The expression of multi-drug resistance (MDR) transporters in CSCs results in drug efflux and decreased intracellular drug concentration (119, 120). The ATP-binding cassette (ABC) transporters encompass 49 members in humans and are organized into seven subfamilies (ABCA-G) (121, 122). Three well-studied members of the family are ABCB1, ABCG2, and ABCC1 (123, 124). Overexpression of ABCG2 is associated with resistance to a large number of chemotherapy agents, such as mitoxantrone, camptothecins and flavopiridol (125). The human breast cancer resistance protein (BCRP/ABCG2), which was derived from the breast cancer cell line Mcf-7, was shown to induce resistance to mitoxantrone (126, 127). However, this resistance could be reverted by the MiR-487a target for the expression of BRCP (126). In ovarian cancer, c-MET/PI3K/AKT pathway activation was shown to induce the expression of BRCP/ABCG2, which is important in doxorubicin resistance (125, 128). ABCB1 is also called P-glycoprotein (P-gp) or multidrug resistance gene 1 (MDR1). In AML, P-gp acts as an adverse prognostic factor for drug resistance (129). It was also found that the absence of miR-298 is related to an over expression of P-gp. The upregulation of miR-298 could reduce the expression P-gp, leading to increased concentration and cytotoxicity of doxorubicin in doxorubicin-resistant breast cells (130). In addition, oncogene kinases such as MEK1/2, ERK1/2, c-Raf, EGF, and FGF, can increase the expression of P-gp and effect drug resistance and therefore may also represent potential targets (131). The ACBC1 transporter is encoded by the MRP1gene. This subfamily plays a role in affecting MDR in lung, bladder, and breast cancer (124). In neuroblastoma, the high expression of MRP1 is associated with a poor outcome and sensitivity to chemotherapy should be regained by targeting MRP1 (132).

Hypoxia influences cancer progression, and therapy resistance, and it leads to poor outcomes. The ROS level is affected by oxygen density. In tumors, hypoxia leads to a low ROS level that in turn can be protective for CSCs and lead to therapy failure (133). HIFs are considered as negative factors for effective tumor therapy. It has been suggested that HIFs influence the pathways that contribute to the quiescence of CSCs, such as cell cycle control via cyclin dependent kinase, metabolic control via pyruvate dependent kinase, anti-apoptosis via BCL-XL, and self-renewal via OCT-4 (134–136). In some reports, HIFs are believed to be related to MDR, such as ABCG2, and to affect drug efficacy. VEGF has also been proven to be induced by hypoxia, leading to chemo/radiotherapy resistance (137, 138). In colon cancer, dual specificity phosphatase-2 (DUSP-2) was suppressed by hypoxic culture, which led to the upregulation of COX-2 expression. DUSP-2 has a negative function in cancer malignancy and COX-2 is closely related to cancer stemness, tumor growth and drug resistance (139).

Anti-apoptosis protein expression in CSCs is another component of therapy resistance. BCL2 and BCL-XL are highly expressed in breast and AML CSCs (136, 140). P53 is a tumor suppressor that is frequently mutated in most human cancers. Due to programs such as inactivation of caspase-9 and protease activating factor1 (Apaf-1), and the activation of gain-of-function, the mutant P53 shows acquisition of dedifferentiation and stemness that leads to drug resistance (141, 142). An altered apoptosis pathway has also been demonstrated to be involved in the formation of drug resistance. The high expression of Bcl-2 due to Notch and Hh signaling pathways translates into docetaxel resistance in prostate CSCs (143). Additionally, Hh signaling pathway activation in AML, especially in CD34^+^ leukemic cell lymphoma, induces the function of anti-apoptosis that lead to chemotherapy (144).

**Figure 3:**
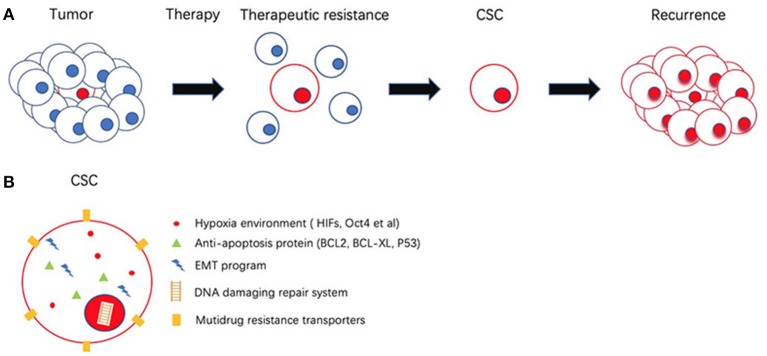
Schematic representation of cancer stem cells (CSCs) and its role in therapeutic resistance. **(A)** Most of the cancer cells are eliminated by therapeutic resistance. CSC could escape from chemotherapy and re-establish tumor. **(B)** CSC possess several mechanisms to achieve therapeutic resistance involves Hypoxia environment, high expression of anti-apoptosis proteins, epithelial mesenchymal transition (EMT), DNA damaging repair system (DDR), multiple drug resistance transporters (MDR).

EMT activities in the CSC environment include wound healing, tissue fibrosis, and carcinoma progression. Non-small lung cancer (NSCLC) treated with Erlotinib targeting EGFR mutation shows more drug resistance due to mesenchymal-like expression. With the reversion of EMT, an elevated epigenetic like the expression of E-cadherin, is associated with sensitivity to the EGFR kinase inhibitor (145). It is generally accepted that the Notch pathway is associated with gemcitabine resistance in PDAC and is regulated by EMT (146). The expression of mesenchymal-like regulators such as Zeb2/SIP1 can protect cells from DNA damage-induced apoptosis in bladder cancer, leading to poor prognosis (147). In non-small lung cancer (NSCLC), EMT is thought to act in the process of quiescence. Overexpressed CSC surface markers, such as ALDH1 and CD133 in NSCLC stem cells are proposed to develop resistance to conventional therapy agents 5-FU (148). CSC-mediated therapeutic resistance relies on different mechanisms. Figure 3 gives insight into illustration of therapeutic resistance driven by CSC and microenvironment.

## Therapeutic Strategies Targeting CSC and their Microenvirements

### CSC Targeting Strategies

The selective targeting CSCs is a promising therapeutic strategy to eliminate the development of human cancer and reduce the risk of recurrence (149). Therapeutic strategies discussed include disrupting the central regulating signaling pathways important for the cell type, targeting specific markers, inhibition of the ABC transporters, manipulating miRNA expression, or inducing the differentiation and apoptosis of CSCs.

Signaling pathways that underlie CSC biology and have been identified as potential targets. Key pathways identified include Sonic hedgehog (Shh)/Patched (Ptch)/Smoothened (Smo), Notch/Delta-like ligand (DLL), CXC chemokine receptor 1-2/CXCL8/FAK, and Wnt pathways. Downstream transcription factors such as β-catenin, STAT3, and Nanog have also been identified as potential clinical targets (150). However, the fact that CSCs and normal stem cells share the expression of many genes and signaling pathways, as well as the redundancy of the regulatory pathways, may effectively limit the efficacy and clinical impact of the therapeutic approaches.

Drugs targeting CSC markers may play an adjunctive role together with surgery, chemotherapy, and radiotherapy (151). CD44, the transmembrane protein that is the receptor for matrix components such as hyaluronic acid selectin, collagen, and osteopontin, has been proven to help treat acute myeloid leukemia (AML) by inhibiting tumor proliferation, and increasing apoptosis (152, 153). CD133, the glycoprotein also known as prominin-1, is another well-known marker on the CSCs surface and it has been reported to be a useful target in cancers with a large CD133 subpopulation (154, 155). In addition, other drugs approved by FDA are also used for targeting CSC markers such as rituximab (anti-CD20) (156), cetuximab (anti-EGFR) (157–159).

The aberrant expression of ABC transporters, which are drug efflux pumps, is a major mechanism of chemoresistance in CSCs cells (160). Three generations of inhibitor drugs have been developed and the fourth generation is underway (49, 160), which should be more selective and less toxic. New technology has been applied to improve therapy efficacy, such as the application of miRNAs targeting specific RNAs or the use of nanomedicines for bypassing efflux pumps. However, currently no inhibitors have been approved for clinical practice. Accumulating studies have reported that different tyrosine kinase inhibitors (TKIs) including erlotinib, lapatinib, imatinib, and nilotinib can reverse drug resistance mediated by ABC transporters (161). The ability to inhibit the multiple regulatory targets of ABC transporters synergisticly, combined with other therapy strategies to overcome chemoresistance in CSCs may represent a promising approach.

CSCs have non-coding RNA profiles that are different from those seen in other cancer cells. Non-coding RNAs act as regulators in maintaining and modifying CSCs properties and functions (162). As such, they represent not only potential drugs but also therapeutic targets for the treatment of CSCs. Accumulating evidence suggests that non-coding RNAs, including microRNA (miRNA) and long non-coding RNA (LncRNA), regulate the stemness of CSCs by acting as suppressors or promoters of pathways that modulate the CSC carcinogenesis, differentiation, and EMT. In breast CSC and CD133 positive pancreatic cancer cells, miR-30 was found to be decreased and inhibited anoikis resistance (163). Three novel LncRNAs including Lnc TCF7, Lnc-b-Catm, and Lnc BRM were reported to sustain the self-renewal of CSCs by regulating the classic signaling pathways related to development and progression of liver CSC (164). Non-coding RNAs are regarded as very useful targets for potential therapeutic strategies due to their limited and selective expression in tumor tissues. MiRNA-based therapeutics are also emerging as tumor treatment options and are currently entering clinical trials (165). For example, a phase 1 clinical trial that targomiRs, minicells loaded with miR-16-based mimic miRNA and targeted to EGFR, is being evaluated in patients with malignant pleural mesothelioma and non-small cell lung cancer (166).

The impairment of apoptosis contributes to cancer development and progression, and the reactivation of apoptosis programs might be useful in the treatment of cancer. It has been reported that targeting TRAIL could cause caspase-8 reactivation, ultimately initiating mitochondrial outer membrane permeabilization and triggering the apoptosis (167). In PACA, JNK inhibition attenuated resistance of TRAIL-induced apoptosis and reduced the self-renewal capacity of CSCs (168). Finally, inhibition of NF-κB by the molecule MG-132, has also been reported to induce cell death (169). Another approach that has been evaluated focused on various means to induce CSCs differentiation. Promising agents are under research currently include the use of retinoic acid and its analogs (ATRA) for the treatment of promyelocytic leukemia (170–172). These may also show utilityto induce differentiation of glioma and breast CSCs. Other molecules such as histone deacetylase inhibitors (HDACI), tyrosine kinase have also been proposed in many CSCs studies (151). Most recent antibody targets in CSC were summarized in Table 1.

**Table 1:** The antibody target in CSC through different mechanisms in different tumors.

**Antigen**	**Targeting mechanism**	**Inhibitor**	**Cancer tested**	**References**
CD44	marker	H90	AML	(142, 143)
CD133	marker	Oxyteracycline FIBPi	Liver Colon	(144, 145)
CD20	Marker	Rituximab	Lymphoma	(146)
EGFR	Marker	Ectuximab	Head and neck Squamous cell Breast Esophagus	(147–149)
ABC transporters		Erlotinib Lapatinib Imatibib Nilotinib	Under test	(151)
targomiRs	EGFR	Clincal phase 1	Malignant pleural mesothelioma Lung	(156)
TRAIL	Apoptosis	JNKi	pancreas	(158)
NF-κB	Apoptosis	MG132	leukemic	(159)

### Targeting the CSC Environment

The tumor environment is comprised of various components including CAFs, immune cells, multipotent stromal cells (MSCs), endothelial and perivascular cells, and their secreted factors including growth factors and cytokines. In addition, this environment is made up of extracellular matrix (ECM) components, and extracellular vesicles, within a prevailing hypoxic region (12). The tumor stroma is thought ot help foster the generation and maintenance of CSCs, protect the tumor from the immune system (173), and contribute to the induction of EMT, leading to enhanced tumor progression, invasion, and recolonization as secondary tumors. Furthermore, CSCs can acquire drug resistance by interacting with niche components in TME. Thus, targeting the TME may represent an effective indirect therapeutic strategy for the treatment of CSCs and for the prevention of drug resistance.

#### Stromal Cells in the CSC Niche

It is recognized that tumor stromal cells can not only provide physical shelter for CSC by limiting drug access, but also promote CSC growth, migration, and metastasis by producing important growth factors, cytokines and chemokines (174). Since the cross-talk between CSCs and stromal cells can stimulate tumor aggressiveness, directly targeting the stromal cells may serve as an alternative therapeutic strategy to treat CSCs (175).

Vascular endothelial cells (ECs), a type of stromal cell in CSC niche, which are required for angiogenesis, can also secrete growth factors and cytokines that enhance the proliferation of cancer cells, and promote the maintenance of CSCs properties in head and neck squamous cell carcinoma (176, 177). Interfering with tumor EC growth and survival could in theory inhibit not only angiogenesis but also the self-replication of CSCs (178). VEGF is a strong proangiogenic factor secreted by cancer cells, that is a well-recognized therapeutic target. Various angiogenic inhibitors have been developed that can also inhibit the self-renewal of CSCs leading to reduced tumor growth. In GBM, the VEGF tyrosine kinase inhibitor—bevacizumab has proven successful in expanding survival time by targeting the perivascular niche (179). Also in GBM, the VEGF-VEGF2-NRP1 axis is seen as an attractive target in order to decrease CD133^+^GBM CSCs (180). Bevacizumab combined with anti-hepatoma-derived growth factor (HDGF) antibody has been shown to suppress CSC populations in NSCLC (181). However, in breast cancer, inhibition of VEGFR may increase CSCs population by inducing hypoxia (182). To address this it may be that use of a VEGFR inhibitor in combination witt HIF inhibition in combination therapy may provide a more effective treatment strategy (183).

CAFs represent the major component of tumor stroma andalso play an important role in cancer therapeutic resistance and radiotherapy resistance. Thus, the direct targeting of CAFs may enhance clinical outcomes. Surface markers of CAFs such as FAP, S1004A, and TEM8 have been directly targeted through administration of sibrotuzumab, 5C3, and ADC, respectively, in various tumor types (184–186). In breast cancer, CAF activation was blocked by inhibition of Hedgehog (Hh) signaling, which also increased the sensitivity of resident CSCs to chemotherapy agents (187). CAFs secret TGF-β, and the inhibition of TGF-β signaling by using LY364947 administered via nanoparticle therapy showed a potent therapeutic effect by disrupting CSC biology (188). PTK7 as a special marker of HNSCC stem cells and is demonstrated to have a close relationship with tumor persistence, metastasis, and recurrence. The use of a PTK7 inhibitor was found to increase the sensitivity of HNSCC to erlotinib (189). In addition to a direct activity on CSCs, the inhibition of important signaling pathways may represent a prospective strategy in tumor treatment. Notch signaling is over-activated in HCC, and is thought to help maintain stemness in liver CSCs by regulating LSD1 deacetylation in CAFs (190). CD10+GPR77+ CAFs were demonstrated to promote cancer stemness and chemoresistance. An antibody against GPR77 was demonstrated to reverse chemoresistance by targeting the CD10+GPR77+ CAF subset in solid tumors, such as in breast, lung cancer (191).

Tumor-associated macrophages (TAMs), feature M2-like characteristics and are important components of TME. TAMs have been demonstrated to promote CSC immunosuppressive traits leading to immune escape (192). It has been suggested that TAMs may represent potential targets for immunotherapy. In inflammatory breast cancer (IBC), tumor cells interact with immune suppressing M2-TAM leading to the production of high levels of IL-8 and CCL18 chemokines that in turn activate STAT3, which induces a CSCs-like phenotype in IBC cells and drives EMT during IBC progression (193). Targeting CXCL8/GRP/STAT3 may represent a therapeutic choice in the treatment of IBC (194). In lung cancer, the Src kinase is associated with metastasis and stemness (195). For example, it has been demonstrated that overexpressing Src in M2-TAMs induces cisplatin resistance in lung cancer. Inhibition of Src using the small molecular agent dasatinib, was found to re-sensitize lung cancer cells to cisplatin (196). CAF derived CXCL12 can help attract monocytes, which display M2 TAM characteristics. Blocking the CXCL12/CXCR4 axis significantly reduced the effect of M2 TME leading to reduced cell proliferation, migration and resistance to vineristine in OSCC chemotherapy (197). A member of the immunoglobulin family, CD47 is found to be overexpressed on the surface of many cancer types. It binds to the signal regulatory protein alpha (SIRPα) to prevent cancer cells from undergoing phagocytosis in the tumor environment (198, 199). Targeting CD47, or interfering with the CD47-SIRPα axis leads to enhanced tumor phagocytosis by macrophages and represents a promising therapeutic strategy to treat CSCs (200–202). It has been shown in HCC that the miRNA 125 delivered via TAM exosomes may significantly suppress the CSC phenotype and limit drug resistance (203). The same function of miRNA125 or TAM exosomes have also been seen in cervical cancer (204), nasopharyngeal cancer (205), and AML (206).

#### Immunotherapy

Immunotherapy is an emerging field and the exact mechanism by which these therapies may abrogate the ability of CSCs to reinitiate tumors is still under investigation. Over the past decade, therapeutic approaches have utilized the cytotoxic T-lymphocyte-associated antigen 4 (CTLA-4) (207) or programmed death 1 (PD-1)/programmed death receptor ligand-1 (PD-L1) (208) blocking antibodies, which have yielded notable response rates and have shown a remarkable clinical response in patients with advanced cancer. Despite the recent successes, the utilization of single antibodies is often limited and leads to poor treatment outcome. To achieve improved immune responses, the use of combination strategies for checkpoint inhibitors with other therapeutics may offer a stronger response against cancer as well as higher recovery rates (209, 210). PD-1 blockade was shown to enhance a specific antitumor efficacy of streptavidin-granulocyte-macrophage-CSF surface-modified bladder cancer stem cells vaccine (211). A recent study showed that targeting CSCs using the CSC-dendritic cell vaccine with CTLA-4 and PD-L1 blockades simultaneously enhanced the eradication of melanoma stem cells in the mouse model (212). In addition, CAR-T cells have produced remarkable antitumor activities in different types of tumors (213, 214). In prostate cancer and NSCLC tumor models, CAR T-cells targeted against EpCAM and EGFR antigens could successfully eradicate CSCs and cancer cells (215, 216). Most recent target factors and chemokines in CSC were summarized in Table 2.

**Table 2:** Target factors and chemokines in different tumors.

**Antigen**	**Derived**	**Inhibitor**	**Cancer tested**	**References**
VEGF	EC	Bevacizumab	GBM Lung	(169, 170) (171)
FAP	CAF	Sibrotuzumab	Colon	(174)
S1004A	CAF/marcophage	5C3	Breast	(175)
TEM8	CAF	ADC	Pancreas Colon Breast	(176)
TGF-β	CAF	LY364947	Under test	(178)
PTK7	CAF		HNSCC	(179)
GPR77	CAF	Anti-GPR77	Lung Breast	(181)
IL8/GRP/ STAT3	TAM	anti IL8/GRP/STAT3 axle	IBC	(183, 184)
Src	TAM	Anti Src/dasatinib	Lung	(186)
CXCL12/ CXCR4	TAM	anti CXCL12/ CXCR4	OSCC	(187)
CD47/ CD47-SIRPα	TAM	Hu5f9-G4	NHL AML ALL	(190–192)
Immune-therapy		PD-1 PD-L1	Balder Melanoma	(201) (202)

## Conclusions and Future Perspectives

Based on the central impact of CSCs on tumor progression with accompanying poor patient outcome, CSCs-targeted therapy approaches have emerged as an important new strategy for future tumor treatments. However, the identification of CSCs remains a challenge. Markers expressed on CSCs may also be displayed by normal stem cells, which may limit the accuracy of CSCs identification and compromise the targeted treatment. In addition, CSCs appear to represent by heterogeneous populations within tumor settings. Therefore, CSC surface markers alone are not broadly reliable for their identification. The best results, absence functional criteria, results from the use of multiple markers which provide a better means of identifying CSC in specific tumor types and may provide information regarding potential drug responsiveness and tumor recurrence.

CSCs have been demonstrated to influence tumor metastasis, immune escape, and drug resistance. Targeting CSCs via their unique signaling pathways, by metabolism reprogramming, hitting the ABC transporters, and even the use of non-coding RNA, represent promising strategies to control tumor progression through CSC-based targeting. However, due to the inherent heterogeneity of CSCs the targeting a single molecule or pathway may not be an effective strategy. Combination therapy may represent the most efficient means for the treatment of CSCs.

In cross-talk with CSCs, the tissue environment plays an important role in the development of tumor metastasis and recurrence. In the TME, CSCs are thought to reside in a special “CSC niche,” which helps maintain their self–renewal and stemness. Immunotherapy represents an important emerging field in tumor therapy. Recent impressive results have been seen in immune targeting of CSCs through the use of PD-1/PDL-1 inhibitors. However, some studies have reported that CSCs are less immunogenic than non-CSCs, and thereby limiting antitumor response to CSCs. CAR-T cells also hold promise in overcoming cancer resistance in different types of tumors. Thus, targeting both CSCs and TME may represent the best option in the anti-cancer approach. Although the interconnected networks between CSCs and TME are complex, and most mechanisms are still obscure, various CSC targeting agents have been developed and successfully tested in several tumor types. In contrast with the single focus of CSCs-targeted therapy, because TME components include different types of stroma cells, cytokines, and growth factors, many of them have proved to be targeted in the eradication of CSCs. However, although there is growing literature in this promising area, the therapeutics of targeting CSCs and their environment are still in its infancy. Research on CSCs and their related environments will provide new targets for the development of anti-tumor strategies. In addition, clarifying the interconnectiveness of CSCs and TME will be important for the design of effective therapeutic approaches. The focus of future trials may include combination therapies that target multiple mechanisms in the tumor. However, this field is still undeveloped, and considerable research will be required to produce viable products. Many important challenges remain, including how to achieve drug selectivity and efficacy, reduce toxicity to normal cells, reduce adverse side effects, and explore new approaches to deliver and keep an effective drug concentration in place. In conclusion, the combined regimen of CSC-targeted therapy together with conventional treatment methods shows great potential and deserves further research.

## Author Contributions

Q-ZD and L-XQ designed and supervised the entire project. H-RS, SW, and S-CY wrote the manuscript. Q-ZD and PN made significant revisions to the manuscript. All authors read and approved the final manuscript.

### Conflict of Interest

The authors declare that the research was conducted in the absence of any commercial or financial relationships that could be construed as a potential conflict of interest.

## References

[1] Torre LA Bray F Siegel RL Ferlay J Lortet-Tieulent J Jemal A. Global cancer statistics, 2012. CA Cancer J Clin. (2015) 65:87–108. 10.3322/caac.21262 25651787

[2] Clarke MF. Self-renewal and solid-tumor stem cells. Biol Blood Marrow Transplant. (2005) 11:14–6. 10.1016/j.bbmt.2004.11.011 15682169

[3] Reya T Morrison SJ Clarke MF Weissman IL. Stem cells, cancer, and cancer stem cells. Nature. (2001) 414:105–11. 10.1038/35102167 11689955

[4] Valent P Bonnet D De Maria R Lapidot T Copland M Melo JV. Cancer stem cell definitions and terminology: the devil is in the details. Nat Rev Cancer. (2012) 12:767–75. 10.1038/nrc3368 23051844

[5] Toledo-Guzman ME Hernandez MI Gomez-Gallegos AA Ortiz-Sanchez E. ALDH as a stem cell marker in solid tumors. Curr Stem Cell Res Ther. (2019) 14:375–88. 10.2174/1574888X13666180810120012 30095061

[6] Leung EL Fiscus RR Tung JW Tin VP Cheng LC Sihoe AD. Non-small cell lung cancer cells expressing CD44 are enriched for stem cell-like properties. PLoS ONE. (2010) 5:e14062. 10.1371/journal.pone.0014062 21124918 PMC2988826

[7] Boesch M Hoflehner E Wolf D Gastl G Sopper S. Harnessing the DNA dye-triggered side population phenotype to detect and purify cancer stem cells from biological samples. J Vis Exp. (2017) e55634. 10.3791/55634 28518118 PMC5607907

[8] Beck B Blanpain C. Unravelling cancer stem cell potential. Nat Rev Cancer. (2013) 13:727–38. 10.1038/nrc3597 24060864

[9] Dean M Fojo T Bates S. Tumour stem cells and drug resistance. Nat Rev Cancer. (2005) 5:275–84. 10.1038/nrc1590 15803154

[10] Peitzsch C Tyutyunnykova A Pantel K Dubrovska A. Cancer stem cells: the root of tumor recurrence and metastases. Semin Cancer Biol. (2017) 44:10–24. 10.1016/j.semcancer.2017.02.011 28257956

[11] Ben-Porath I Thomson MW Carey VJ Ge R Bell GW Regev A. An embryonic stem cell-like gene expression signature in poorly differentiated aggressive human tumors. Nat Genet. (2008) 40:499–507. 10.1038/ng.127 18443585 PMC2912221

[12] Plaks V Kong N Werb Z. The cancer stem cell niche: how essential is the niche in regulating stemness of tumor cells? Cell Stem Cell. (2015) 16:225–38. 10.1016/j.stem.2015.02.015 25748930 PMC4355577

[13] Gottesman MM Pastan I. Biochemistry of multidrug resistance mediated by the multidrug transporter. Annu Rev Biochem. (1993) 62:385–427. 10.1146/annurev.bi.62.070193.002125 8102521

[14] Marusyk A Polyak K. Tumor heterogeneity: causes and consequences. Biochim Biophys Acta. (2010) 1805:105–17. 10.1016/j.bbcan.2009.11.002 19931353 PMC2814927

[15] Kleffel S Schatton T. Tumor dormancy and cancer stem cells: two sides of the same coin? Adv Exp Med Biol. (2013) 734:145–79. 10.1007/978-1-4614-1445-2_8 23143979

[16] Cai Z Cao Y Luo Y Hu H Ling H. Signalling mechanism(s) of epithelial-mesenchymal transition and cancer stem cells in tumour therapeutic resistance. Clin Chim Acta. (2018) 483:156–63. 10.1016/j.cca.2018.04.033 29709449

[17] Takebe N Miele L Harris PJ Jeong W Bando H Kahn M. Targeting Notch, Hedgehog, and Wnt pathways in cancer stem cells: clinical update. Nat Rev Clin Oncol. (2015) 12:445–64. 10.1038/nrclinonc.2015.61 25850553 PMC4520755

[18] Visvader JE Lindeman GJ. Cancer stem cells in solid tumours: accumulating evidence and unresolved questions. Nat Rev Cancer. (2008) 8:755–68. 10.1038/nrc2499 18784658

[19] Tirino V Desiderio V Paino F De Rosa A Papaccio F La Noce M. Cancer stem cells in solid tumors: an overview and new approaches for their isolation and characterization. FASEB J. (2013) 27:13–24. 10.1096/fj.12-218222 23024375

[20] Bonnet D Dick JE. Human acute myeloid leukemia is organized as a hierarchy that originates from a primitive hematopoietic cell. Nat Med. (1997) 3:730–7. 10.1038/nm0797-730 9212098

[21] Xia P. Surface markers of cancer stem cells in solid tumors. Curr Stem Cell Res Ther. (2014) 9:102–11. 10.2174/1574888X09666131217003709 24359139

[22] Bach P Abel T Hoffmann C Gal Z Braun G Voelker I. Specific elimination of CD133+ tumor cells with targeted oncolytic measles virus. Cancer Res. (2013) 73:865–74. 10.1158/0008-5472.CAN-12-2221 23293278

[23] Dianat-Moghadam H Heidarifard M Jahanban-Esfahlan R Panahi Y Hamishehkar H Pouremamali F. Cancer stem cells-emanated therapy resistance: Implications for liposomal drug delivery systems. J Control Release. (2018) 288:62–83. 10.1016/j.jconrel.2018.08.043 30184466

[24] Heiler S Wang Z Zoller M. Pancreatic cancer stem cell markers and exosomes - the incentive push. World J Gastroenterol. (2016) 22:5971–6007. 10.3748/wjg.v22.i26.5971 27468191 PMC4948278

[25] Batlle E Clevers H. Cancer stem cells revisited. Nat Med. (2017) 23:1124–34. 10.1038/nm.4409 28985214

[26] Comoglio PM Trusolino L Boccaccio C. Known and novel roles of the MET oncogene in cancer: a coherent approach to targeted therapy. Nat Rev Cancer. (2018) 18:341–58. 10.1038/s41568-018-0002-y 29674709

[27] Grosse-Gehling P Fargeas CA Dittfeld C Garbe Y Alison MR Corbeil D. CD133 as a biomarker for putative cancer stem cells in solid tumours: limitations, problems and challenges. J Pathol. (2013) 229:355–78. 10.1002/path.4086 22899341

[28] Collins AT Berry PA Hyde C Stower MJ Maitland NJ. Prospective identification of tumorigenic prostate cancer stem cells. Cancer Res. (2005) 65:10946–51. 10.1158/0008-5472.CAN-05-2018 16322242

[29] Li C Lee CJ Simeone DM. Identification of human pancreatic cancer stem cells. Methods Mol Biol. (2009) 568:161–73. 10.1007/978-1-59745-280-9_10 19582426

[30] Singh SK Hawkins C Clarke ID Squire JA Bayani J Hide T. Identification of human brain tumour initiating cells. Nature. (2004) 432:396–401. 10.1038/nature03128 15549107

[31] Choi YJ Park SJ Park YS Park HS Yang KM Heo K. EpCAM peptide-primed dendritic cell vaccination confers significant anti-tumor immunity in hepatocellular carcinoma cells. PLoS ONE. (2018) 13:e0190638. 10.1371/journal.pone.0190638 29298343 PMC5752035

[32] O'Brien CA Pollett A Gallinger S Dick JE. A human colon cancer cell capable of initiating tumour growth in immunodeficient mice. Nature. (2007) 445:106–10. 10.1038/nature05372 17122772

[33] Curley MD Therrien VA Cummings CL Sergent PA Koulouris CR Friel AM. CD133 expression defines a tumor initiating cell population in primary human ovarian cancer. Stem Cells. (2009) 27:2875–83. 10.1002/stem.236 19816957

[34] Boiko AD Razorenova OV van de Rijn M Swetter SM Johnson DL Ly DP. Human melanoma-initiating cells express neural crest nerve growth factor receptor CD271. Nature. (2010) 466:133–7. 10.1038/nature09161 20596026 PMC2898751

[35] Zhang WC Shyh-Chang N Yang H Rai A Umashankar S Ma S. Glycine decarboxylase activity drives non-small cell lung cancer tumor-initiating cells and tumorigenesis. Cell. (2012) 148:259–72. 10.1016/j.cell.2011.11.050 22225612

[36] Wicha MS Liu S Dontu G Cancer stem cells: an old idea–a paradigm shift. Cancer Res. (2006) 66:1883–90. Discussion 1895–6. 10.1158/0008-5472.CAN-05-3153 16488983

[37] Shmelkov SV Butler JM Hooper AT Hormigo A Kushner J Milde T CD133 expression is not restricted to stem cells, and both CD133+ and CD133- metastatic colon cancer cells initiate tumors. J Clin Invest. (2008) 118:2111–20. 10.1172/JCI34401 18497886 PMC2391278

[38] Patriarca C Macchi RM Marschner AK Mellstedt H. Epithelial cell adhesion molecule expression (CD326) in cancer: a short review. Cancer Treat Rev. (2012) 38:68–75. 10.1016/j.ctrv.2011.04.002 21576002

[39] Yamashita T Ji J Budhu A Forgues M Yang W Wang HY. EpCAM-positive hepatocellular carcinoma cells are tumor-initiating cells with stem/progenitor cell features. Gastroenterology. (2009) 136:1012–24. 10.1053/j.gastro.2008.12.004 19150350 PMC2828822

[40] Sundberg M Jansson L Ketolainen J Pihlajamaki H Suuronen R Skottman H. CD marker expression profiles of human embryonic stem cells and their neural derivatives, determined using flow-cytometric analysis, reveal a novel CD marker for exclusion of pluripotent stem cells. Stem Cell Res. (2009) 2:113–24. 10.1016/j.scr.2008.08.001 19383417

[41] Al-Hajj M Wicha MS Benito-Hernandez A Morrison SJ Clarke MF. Prospective identification of tumorigenic breast cancer cells. Proc Natl Acad Sci USA. (2003) 100:3983–8. 10.1073/pnas.0530291100 12629218 PMC153034

[42] Zhang C Li C He F Cai Y Yang H. Identification of CD44+CD24+ gastric cancer stem cells. J Cancer Res Clin Oncol. (2011) 137:1679–86. 10.1007/s00432-011-1038-5 21882047 PMC11828146

[43] Ou X O'Leary HA Broxmeyer HE. Implications of DPP4 modification of proteins that regulate stem/progenitor and more mature cell types. Blood. (2013) 122:161–9. 10.1182/blood-2013-02-487470 23637126 PMC3709652

[44] Pang R Law WL Chu AC Poon JT Lam CS Chow AK. A subpopulation of CD26+ cancer stem cells with metastatic capacity in human colorectal cancer. Cell Stem Cell. (2010) 6:603–15. 10.1016/j.stem.2010.04.001 20569697

[45] Bai X Ni J Beretov J Graham P Li Y. Cancer stem cell in breast cancer therapeutic resistance. Cancer Treat Rev. (2018) 69:152–63. 10.1016/j.ctrv.2018.07.004 30029203

[46] Yao T Wu Z Liu Y Rao Q Lin Z. Aldehyde dehydrogenase 1 (ALDH1) positivity correlates with poor prognosis in cervical cancer. J Int Med Res. (2014) 42:1038–42. 10.1177/0300060514527060 24827824

[47] Christgen M Ballmaier M Bruchhardt H von Wasielewski R Kreipe H Lehmann U. Identification of a distinct side population of cancer cells in the Cal-51 human breast carcinoma cell line. Mol Cell Biochem. (2007) 306:201–12. 10.1007/s11010-007-9570-y 17660947

[48] Zhou BB Zhang H Damelin M Geles KG Grindley JC Dirks PB. Tumour-initiating cells: challenges and opportunities for anticancer drug discovery. Nat Rev Drug Discov. (2009) 8:806–23. 10.1038/nrd2137 19794444

[49] Su YJ Chang YW Lin WH Liang CL Lee JL. An aberrant nuclear localization of E-cadherin is a potent inhibitor of Wnt/beta-catenin-elicited promotion of the cancer stem cell phenotype. Oncogenesis. (2015) 4:e157. 10.1038/oncsis.2015.17 26075748 PMC4491612

[50] Barnawi R Al-Khaldi S Colak D Tulbah A Al-Tweigeri T Fallatah M. beta1 Integrin is essential for fascin-mediated breast cancer stem cell function and disease progression. Int J Cancer. (2019) 145:830–41. 10.1002/ijc.32183 30719702 PMC6593770

[51] Wang SJ Bourguignon LY. Role of hyaluronan-mediated CD44 signaling in head and neck squamous cell carcinoma progression and chemoresistance. Am J Pathol. (2011) 178:956–63. 10.1016/j.ajpath.2010.11.077 21356346 PMC3069910

[52] Screaton GR Bell MV Jackson DG Cornelis FB Gerth U Bell JI. Genomic structure of DNA encoding the lymphocyte homing receptor CD44 reveals at least 12 alternatively spliced exons. Proc Natl Acad Sci USA. (1992) 89:12160–4. 10.1073/pnas.89.24.12160 1465456 PMC50718

[53] Screaton GR Bell MV Bell JI Jackson DG. The identification of a new alternative exon with highly restricted tissue expression in transcripts encoding the mouse Pgp-1 (CD44) homing receptor. Comparison of all 10 variable exons between mouse, human, and rat. J Biol Chem. (1993) 268:12235–8. 8509359

[54] Kuhn S Koch M Nubel T Ladwein M Antolovic D Klingbeil P. A complex of EpCAM, claudin-7, CD44 variant isoforms, and tetraspanins promotes colorectal cancer progression. Mol Cancer Res. (2007) 5:553–67. 10.1158/1541-7786.MCR-06-0384 17579117

[55] Zhao S He JL Qiu ZX Chen NY Luo Z Chen BJ. Prognostic value of CD44 variant exon 6 expression in non-small cell lung cancer: a meta-analysis. Asian Pac J Cancer Prev. (2014) 15:6761–6. 10.7314/APJCP.2014.15.16.6761 25169522

[56] Jiang H Zhao W Shao W. Prognostic value of CD44 and CD44v6 expression in patients with non-small cell lung cancer: meta-analysis. Tumour Biol. (2014) 35:7383–9. 10.1007/s13277-014-2150-3 24913707

[57] Kaufmann M Heider KH Sinn HP von Minckwitz G Ponta H Herrlich P. CD44 variant exon epitopes in primary breast cancer and length of survival. Lancet. (1995) 345:615–9. 10.1016/S0140-6736(95)90521-9 7534855

[58] Rys J Kruczak A Lackowska B Jaszcz-Gruchala A Brandys A Stelmach A. The role of CD44v3 expression in female breast carcinomas. Pol J Pathol. (2003) 54:243–7. 14998292

[59] Frank NY Pendse SS Lapchak PH Margaryan A Shlain D Doeing C. Regulation of progenitor cell fusion by ABCB5 P-glycoprotein, a novel human ATP-binding cassette transporter. J Biol Chem. (2003) 278:47156–65. 10.1074/jbc.M308700200 12960149

[60] Jongkhajornpong P Nakamura T Sotozono C Nagata M Inatomi T Kinoshita S. Elevated expression of ABCB5 in ocular surface squamous neoplasia. Sci Rep. (2016) 6:20541. 10.1038/srep20541 26843453 PMC4740799

[61] Chartrain M Riond J Stennevin A Vandenberghe I Gomes B Lamant L. Melanoma chemotherapy leads to the selection of ABCB5-expressing cells. PLoS ONE. (2012) 7:e36762. 10.1371/journal.pone.0036762 22675422 PMC3360047

[62] Schatton T Murphy GF Frank NY Yamaura K Waaga-Gasser AM Gasser M. Identification of cells initiating human melanomas. Nature. (2008) 451:345–9. 10.1038/nature06489 18202660 PMC3660705

[63] Linley AJ Mathieu MG Miles AK Rees RC McArdle SE Regad T. The helicase HAGE expressed by malignant melanoma-initiating cells is required for tumor cell proliferation *in vivo*. J Biol Chem. (2012) 287:13633–43. 10.1074/jbc.M111.308973 22393060 PMC3340133

[64] Schatton T Schutte U Frank NY Zhan Q Hoerning A Robles SC. Modulation of T-cell activation by malignant melanoma initiating cells. Cancer Res. (2010) 70:697–708. 10.1158/0008-5472.CAN-09-1592 20068175 PMC2883769

[65] Schmohl JU Vallera DA. CD133, selectively targeting the root of cancer. Toxins. (2016) 8:E165. 10.3390/toxins8060165 27240402 PMC4926132

[66] Hanahan D Weinberg RA. Hallmarks of cancer: the next generation. Cell. (2011) 144:646–74. 10.1016/j.cell.2011.02.013 21376230

[67] Pistollato F Abbadi S Rampazzo E Persano L Della Puppa A Frasson C. Intratumoral hypoxic gradient drives stem cells distribution and MGMT expression in glioblastoma. Stem Cells. (2010) 28:851–62. 10.1002/stem.415 20309962

[68] Heddleston JM Li Z McLendon RE Hjelmeland AB Rich JN. The hypoxic microenvironment maintains glioblastoma stem cells and promotes reprogramming towards a cancer stem cell phenotype. Cell Cycle. (2009) 8:3274–84. 10.4161/cc.8.20.9701 19770585 PMC2825672

[69] Ye J Wu D Wu P Chen Z Huang J. The cancer stem cell niche: cross talk between cancer stem cells and their microenvironment. Tumour Biol. (2014) 35:3945–51. 10.1007/s13277-013-1561-x 24420150

[70] Campos-Sanchez E Cobaleda C Tumoral reprogramming: plasticity takes a walk on the wild side Biochim Biophys Acta. (2015) 1849:436–47. 10.1016/j.bbagrm.2014.07.003 25038581

[71] Hjelmeland AB Wu Q Heddleston JM Choudhary GS MacSwords J Lathia JD. Acidic stress promotes a glioma stem cell phenotype. Cell Death Differ. (2011) 18:829–40. 10.1038/cdd.2010.150 21127501 PMC3095828

[72] Balkwill FR Capasso M Hagemann T. The tumor microenvironment at a glance. J Cell Sci. (2012) 125:5591–6. 10.1242/jcs.116392 23420197

[73] Charles N Ozawa T Squatrito M Bleau AM Brennan CW Hambardzumyan D. Perivascular nitric oxide activates notch signaling and promotes stem-like character in PDGF-induced glioma cells. Cell Stem Cell. (2010) 6:141–52. 10.1016/j.stem.2010.01.001 20144787 PMC3818090

[74] D'Ippolito G Diabira S Howard GA Roos BA Schiller PC. Low oxygen tension inhibits osteogenic differentiation and enhances stemness of human MIAMI cells. Bone. (2006) 39:513–22. 10.1016/j.bone.2006.02.061 16616713

[75] Tang BZ Zhao FY Qu Y Mu DZ. [Hypoxia-inducible factor-1alpha: a promising target for tumor therapy]. Ai Zheng. (2009) 28:775–82. 10.5732/cjc.008.10770 19624909

[76] Gidekel S Pizov G Bergman Y Pikarsky E. Oct-3/4 is a dose-dependent oncogenic fate determinant. Cancer Cell. (2003) 4:361–70. 10.1016/S1535-6108(03)00270-8 14667503

[77] Masui S Nakatake Y Toyooka Y Shimosato D Yagi R Takahashi K. Pluripotency governed by Sox2 via regulation of Oct3/4 expression in mouse embryonic stem cells. Nat Cell Biol. (2007) 9:625–35. 10.1038/ncb1589 17515932

[78] Yan ZY Sun XC. [LincRNA-ROR functions as a ceRNA to regulate Oct4, Sox2, and Nanog expression by sponging miR-145 and its effect on biologic characteristics of colonic cancer stem cells]. Zhonghua Bing Li Xue Za Zhi. (2018) 47:284–90. 10.3760/cma.j.issn.0529-5807.2018.04.011 29690669

[79] Yan B Jiang Z Cheng L Chen K Zhou C Sun L. Paracrine HGF/c-MET enhances the stem cell-like potential and glycolysis of pancreatic cancer cells via activation of YAP/HIF-1alpha. Exp Cell Res. (2018) 371:63–71. 10.1016/j.yexcr.2018.07.041 30056064

[80] Roche TE Baker JC Yan X Hiromasa Y Gong X Peng T. Distinct regulatory properties of pyruvate dehydrogenase kinase and phosphatase isoforms. Prog Nucleic Acid Res Mol Biol. (2001) 70:33–75. 10.1016/S0079-6603(01)70013-X 11642366

[81] Dupuy F Tabaries S Andrzejewski S Dong Z Blagih J Annis MG. PDK1-dependent metabolic reprogramming dictates metastatic potential in breast cancer. Cell Metab. (2015) 22:577–89. 10.1016/j.cmet.2015.08.007 26365179

[82] Peng F Wang JH Fan WJ Meng YT Li MM Li TT Glycolysis gatekeeper PDK1 reprograms breast cancer stem cells under hypoxia. Oncogene. (2018) 37:1119 10.1038/onc.2017.407 29251717 PMC5851083

[83] Ye XQ Li Q Wang GH Sun FF Huang GJ Bian XW. Mitochondrial and energy metabolism-related properties as novel indicators of lung cancer stem cells. Int J Cancer. (2011) 129:820–31. 10.1002/ijc.25944 21520032

[84] Janiszewska M Suva ML Riggi N Houtkooper RH Auwerx J Clement-Schatlo V. Imp2 controls oxidative phosphorylation and is crucial for preserving glioblastoma cancer stem cells. Genes Dev. (2012) 26:1926–44. 10.1101/gad.188292.112 22899010 PMC3435496

[85] Sancho P Burgos-Ramos E Tavera A Bou Kheir T Jagust P Schoenhals M. MYC/PGC-1alpha balance determines the metabolic phenotype and plasticity of pancreatic cancer stem cells. Cell Metab. (2015) 22:590–605. 10.1016/j.cmet.2015.08.015 26365176

[86] Chen WJ Ho CC Chang YL Chen HY Lin CA Ling TY. Cancer-associated fibroblasts regulate the plasticity of lung cancer stemness via paracrine signalling. Nat Commun. (2014) 5:3472. 10.1038/ncomms4472 24668028

[87] Tsuyada A Chow A Wu J Somlo G Chu P Loera S. CCL2 mediates cross-talk between cancer cells and stromal fibroblasts that regulates breast cancer stem cells. Cancer Res. (2012) 72:2768–79. 10.1158/0008-5472.CAN-11-3567 22472119 PMC3367125

[88] Kinugasa Y Matsui T Takakura N. CD44 expressed on cancer-associated fibroblasts is a functional molecule supporting the stemness and drug resistance of malignant cancer cells in the tumor microenvironment. Stem Cells. (2014) 32:145–56. 10.1002/stem.1556 24395741

[89] Zhao XL Lin Y Jiang J Tang Z Yang S Lu L. High-mobility group box 1 released by autophagic cancer-associated fibroblasts maintains the stemness of luminal breast cancer cells. J Pathol. (2017) 243:376–89. 10.1002/path.4958 28802057 PMC8171497

[90] Bouras T Pal B Vaillant F Harburg G Asselin-Labat ML Oakes SR. Notch signaling regulates mammary stem cell function and luminal cell-fate commitment. Cell Stem Cell. (2008) 3:429–41. 10.1016/j.stem.2008.08.001 18940734

[91] Liu C Liu L Chen X Cheng J Zhang H Zhang C. LSD1 stimulates cancer-associated fibroblasts to drive Notch3-dependent self-renewal of liver cancer stem-like cells. Cancer Res. (2018) 78:938–49. 10.1158/0008-5472.CAN-17-1236 29259010

[92] Jung Y Kim JK Shiozawa Y Wang J Mishra A Joseph J. Recruitment of mesenchymal stem cells into prostate tumours promotes metastasis. Nat Commun. (2013) 4:1795. 10.1038/ncomms2766 23653207 PMC3649763

[93] Nair N Calle AS Zahra MH Prieto-Vila M Oo AKK Hurley L. A cancer stem cell model as the point of origin of cancer-associated fibroblasts in tumor microenvironment. Sci Rep. (2017) 7:6838. 10.1038/s41598-017-07144-5 28754894 PMC5533745

[94] Valenti G Quinn HM Heynen G Lan L Holland JD Vogel R. Cancer stem cells regulate cancer-associated fibroblasts via activation of hedgehog signaling in mammary gland tumors. Cancer Res. (2017) 77:2134–47. 10.1158/0008-5472.CAN-15-3490 28202523

[95] Diehn M Cho RW Lobo NA Kalisky T Dorie MJ Kulp AN. Association of reactive oxygen species levels and radioresistance in cancer stem cells. Nature. (2009) 458:780–3. 10.1038/nature07733 19194462 PMC2778612

[96] Li X Lewis MT Huang J Gutierrez C Osborne CK Wu MF. Intrinsic resistance of tumorigenic breast cancer cells to chemotherapy. J Natl Cancer Inst. (2008) 100:672–9. 10.1093/jnci/djn123 18445819

[97] Chen T Dent SY. Chromatin modifiers and remodellers: regulators of cellular differentiation. Nat Rev Genet. (2014) 15:93–106. 10.1038/nrg3607 24366184 PMC3999985

[98] Meacham CE Morrison SJ. Tumour heterogeneity and cancer cell plasticity. Nature. (2013) 501:328–37. 10.1038/nature12624 24048065 PMC4521623

[99] Peitzsch C Kurth I Kunz-Schughart L Baumann M Dubrovska A. Discovery of the cancer stem cell related determinants of radioresistance. Radiother Oncol. (2013) 108:378–87. 10.1016/j.radonc.2013.06.003 23830195

[100] Sosa MS Bragado P Aguirre-Ghiso JA. Mechanisms of disseminated cancer cell dormancy: an awakening field. Nat Rev Cancer. (2014) 14:611–22. 10.1038/nrc3793 25118602 PMC4230700

[101] Crea F Nur Saidy NR Collins CC Wang Y. The epigenetic/noncoding origin of tumor dormancy. Trends Mol Med. (2015) 21:206–11. 10.1016/j.molmed.2015.02.005 25771096

[102] Morrison R Schleicher SM Sun Y Niermann KJ Kim S Spratt DE. Targeting the mechanisms of resistance to chemotherapy and radiotherapy with the cancer stem cell hypothesis. J Oncol. (2011) 2011:941876. 10.1155/2011/941876 20981352 PMC2958340

[103] Alison MR Lim SM Nicholson LJ. Cancer stem cells: problems for therapy? J Pathol. (2011) 223:147–61. 10.1002/path.2793 21125672

[104] Raguz S Yague E. Resistance to chemotherapy: new treatments and novel insights into an old problem. Br J Cancer. (2008) 99:387–91. 10.1038/sj.bjc.6604510 18665178 PMC2527800

[105] Signore M Ricci-Vitiani L De Maria R. Targeting apoptosis pathways in cancer stem cells. Cancer Lett. (2013) 332:374–82. 10.1016/j.canlet.2011.01.013 21315505

[106] Skvortsov S Debbage P Lukas P Skvortsova I. Crosstalk between DNA repair and cancer stem cell (CSC) associated intracellular pathways. Semin Cancer Biol. (2015) 31:36–42. 10.1016/j.semcancer.2014.06.002 24954010

[107] Fishel ML Jiang Y Rajeshkumar NV Scandura G Sinn AL He Y. Impact of APE1/Ref-1 redox inhibition on pancreatic tumor growth. Mol Cancer Ther. (2011) 10:1698–708. 10.1158/1535-7163.MCT-11-0107 21700832 PMC3170439

[108] Wang K Zhang T Dong Q Nice EC Huang C Wei Y. Redox homeostasis: the linchpin in stem cell self-renewal and differentiation. Cell Death Dis. (2013) 4:e537. 10.1038/cddis.2013.50 23492768 PMC3613828

[109] Blanpain C Mohrin M Sotiropoulou PA Passegue E. DNA-damage response in tissue-specific and cancer stem cells. Cell Stem Cell. (2011) 8:16–29. 10.1016/j.stem.2010.12.012 21211780

[110] Skvortsova I Skvortsov S Stasyk T Raju U Popper BA Schiestl B. Intracellular signaling pathways regulating radioresistance of human prostate carcinoma cells. Proteomics. (2008) 8:4521–33. 10.1002/pmic.200800113 18821526

[111] Williams GJ Lees-Miller SP Tainer JA. Mre11-Rad50-Nbs1 conformations and the control of sensing, signaling, and effector responses at DNA double-strand breaks. DNA Repair. (2010) 9:1299–306. 10.1016/j.dnarep.2010.10.001 21035407 PMC3008338

[112] Kuroda S Urata Y Fujiwara T. Ataxia-telangiectasia mutated and the Mre11-Rad50-NBS1 complex: promising targets for radiosensitization. Acta Med Okayama. (2012) 66:83–92. 10.18926/AMO/48258 22525466

[113] Anuranjani Bala M. Concerted action of Nrf2-ARE pathway, MRN complex, HMGB1 and inflammatory cytokines - implication in modification of radiation damage. Redox Biol. (2014) 2:832–46. 10.1016/j.redox.2014.02.008 25009785 PMC4085347

[114] Altan B Yokobori T Ide M Bai T Yanoma T Kimura A. High expression of MRE11-RAD50-NBS1 is associated with poor prognosis and chemoresistance in gastric cancer. Anticancer Res. (2016) 36:5237–47. 10.21873/anticanres.11094 27798884

[115] Zhao Y Chen S. Targeting DNA Double-Strand Break (DSB) Repair to counteract tumor radio-resistance. Curr Drug Targets. (2019) 20:891–902. 10.2174/1389450120666190222181857 30806313

[116] Zona S Bella L Burton MJ Nestal de Moraes G Lam EW. FOXM1: an emerging master regulator of DNA damage response and genotoxic agent resistance. Biochim Biophys Acta. (2014) 1839:1316–22. 10.1016/j.bbagrm.2014.09.016 25287128 PMC4316173

[117] Tripathi K Mani C Barnett R Nalluri S Bachaboina L Rocconi RP. Gli1 protein regulates the S-phase checkpoint in tumor cells via Bid protein, and its inhibition sensitizes to DNA topoisomerase 1 inhibitors. J Biol Chem. (2014) 289:31513–25. 10.1074/jbc.M114.606483 25253693 PMC4223349

[118] Jackson SP Bartek J. The DNA-damage response in human biology and disease. Nature. (2009) 461:1071–8. 10.1038/nature08467 19847258 PMC2906700

[119] Kapse-Mistry S Govender T Srivastava R Yergeri M. Nanodrug delivery in reversing multidrug resistance in cancer cells. Front Pharmacol. (2014) 5:159. 10.3389/fphar.2014.00159 25071577 PMC4090910

[120] Ho EA Piquette-Miller M. Regulation of multidrug resistance by pro-inflammatory cytokines. Curr Cancer Drug Targets. (2006) 6:295–311. 10.2174/156800906777441753 16848721

[121] Wilson BJ Schatton T Zhan Q Gasser M Ma J Saab KR. ABCB5 identifies a therapy-refractory tumor cell population in colorectal cancer patients. Cancer Res. (2011) 71:5307–16. 10.1158/0008-5472.CAN-11-0221 21652540 PMC3395026

[122] Shervington A Lu C. Expression of multidrug resistance genes in normal and cancer stem cells. Cancer Invest. (2008) 26:535–42. 10.1080/07357900801904140 18568776

[123] Shukla S Ohnuma S Ambudkar SV. Improving cancer chemotherapy with modulators of ABC drug transporters. Curr Drug Targets. (2011) 12:621–30. 10.2174/138945011795378540 21039338 PMC3401946

[124] Tiwari AK Sodani K Dai CL Ashby CR Jr Chen ZS. Revisiting the ABCs of multidrug resistance in cancer chemotherapy. Curr Pharm Biotechnol. (2011) 12:570–94. 10.2174/138920111795164048 21118094

[125] Robey RW Polgar O Deeken J To KW Bates SE. ABCG2: determining its relevance in clinical drug resistance. Cancer Metastasis Rev. (2007) 26:39–57. 10.1007/s10555-007-9042-6 17323127

[126] Ma MT He M Wang Y Jiao XY Zhao L Bai XF. MiR-487a resensitizes mitoxantrone (MX)-resistant breast cancer cells (MCF-7/MX) to MX by targeting breast cancer resistance protein (BCRP/ABCG2). Cancer Lett. (2013) 339:107–15. 10.1016/j.canlet.2013.07.016 23879965

[127] Ni Z Bikadi Z Rosenberg MF Mao Q. Structure and function of the human breast cancer resistance protein (BCRP/ABCG2). Curr Drug Metab. (2010) 11:603–17. 10.2174/138920010792927325 20812902 PMC2950214

[128] Vadlapatla RK Vadlapudi AD Pal D Mitra AK. Mechanisms of drug resistance in cancer chemotherapy: coordinated role and regulation of efflux transporters and metabolizing enzymes. Curr Pharm Des. (2013) 19:7126–40. 10.2174/13816128113199990493 23829373

[129] Schaich M Soucek S Thiede C Ehninger G Illmer T Group SAS. MDR1 and MRP1 gene expression are independent predictors for treatment outcome in adult acute myeloid leukaemia. Br J Haematol. (2005) 128:324–32. 10.1111/j.1365-2141.2004.05319.x 15667534

[130] Bao L Hazari S Mehra S Kaushal D Moroz K Dash S. Increased expression of P-glycoprotein and doxorubicin chemoresistance of metastatic breast cancer is regulated by miR-298. Am J Pathol. (2012) 180:2490–503. 10.1016/j.ajpath.2012.02.024 22521303 PMC3378910

[131] Katayama K Yoshioka S Tsukahara S Mitsuhashi J Sugimoto Y. Inhibition of the mitogen-activated protein kinase pathway results in the down-regulation of P-glycoprotein. Mol Cancer Ther. (2007) 6:2092–102. 10.1158/1535-7163.MCT-07-0148 17620438

[132] Haber M Smith J Bordow SB Flemming C Cohn SL London WB. Association of high-level MRP1 expression with poor clinical outcome in a large prospective study of primary neuroblastoma. J Clin Oncol. (2006) 24:1546–53. 10.1200/JCO.2005.01.6196 16575006

[133] Hockel M Vaupel P. Tumor hypoxia: definitions and current clinical, biologic, and molecular aspects. J Natl Cancer Inst. (2001) 93:266–76. 10.1093/jnci/93.4.266 11181773

[134] Cheung TH Rando TA. Molecular regulation of stem cell quiescence. Nat Rev Mol Cell Biol. (2013) 14:329–40. 10.1038/nrm3591 23698583 PMC3808888

[135] Schoning JP Monteiro M Gu W. Drug resistance and cancer stem cells: the shared but distinct roles of hypoxia-inducible factors HIF1alpha and HIF2alpha. Clin Exp Pharmacol Physiol. (2017) 44:153–61. 10.1111/1440-1681.12693 27809360

[136] Konopleva M Zhao S Hu W Jiang S Snell V Weidner D. The anti-apoptotic genes Bcl-X(L) and Bcl-2 are over-expressed and contribute to chemoresistance of non-proliferating leukaemic CD34+ cells. Br J Haematol. (2002) 118:521–34. 10.1046/j.1365-2141.2002.03637.x 12139741

[137] Harada H. How can we overcome tumor hypoxia in radiation therapy? J Radiat Res. (2011) 52:545–56. 10.1269/jrr.11056 21952313

[138] Wilson WR Hay MP. Targeting hypoxia in cancer therapy. Nat Rev Cancer. (2011) 11:393–410. 10.1038/nrc3064 21606941

[139] Hou PC Li YH Lin SC Lin SC Lee JC Lin BW. Hypoxia-induced downregulation of DUSP-2 phosphatase drives colon cancer stemness. Cancer Res. (2017) 77:4305–16. 10.1158/0008-5472.CAN-16-2990 28652251

[140] Madjd Z Mehrjerdi AZ Sharifi AM Molanaei S Shahzadi SZ Asadi-Lari M. CD44+ cancer cells express higher levels of the anti-apoptotic protein Bcl-2 in breast tumours. Cancer Immun. (2009) 9:4. 19385591 PMC2935767

[141] Sarig R Rivlin N Brosh R Bornstein C Kamer I Ezra O. Mutant p53 facilitates somatic cell reprogramming and augments the malignant potential of reprogrammed cells. J Exp Med. (2010) 207:2127–40. 10.1084/jem.20100797 20696700 PMC2947075

[142] Soengas MS Alarcon RM Yoshida H Giaccia AJ Hakem R Mak TW. Apaf-1 and caspase-9 in p53-dependent apoptosis and tumor inhibition. Science. (1999) 284:156–9. 10.1126/science.284.5411.156 10102818

[143] Domingo-Domenech J Vidal SJ Rodriguez-Bravo V Castillo-Martin M Quinn SA Rodriguez-Barrueco R. Suppression of acquired docetaxel resistance in prostate cancer through depletion of notch- and hedgehog-dependent tumor-initiating cells. Cancer Cell. (2012) 22:373–88. 10.1016/j.ccr.2012.07.016 22975379 PMC5989708

[144] Kobune M Takimoto R Murase K Iyama S Sato T Kikuchi S. Drug resistance is dramatically restored by hedgehog inhibitors in CD34+ leukemic cells. Cancer Sci. (2009) 100:948–55. 10.1111/j.1349-7006.2009.01111.x 19245435 PMC11158794

[145] Witta SE Gemmill RM Hirsch FR Coldren CD Hedman K Ravdel L. Restoring E-cadherin expression increases sensitivity to epidermal growth factor receptor inhibitors in lung cancer cell lines. Cancer Res. (2006) 66:944–50. 10.1158/0008-5472.CAN-05-1988 16424029

[146] Hindriksen S Bijlsma MF. Cancer stem cells, EMT, and developmental pathway activation in pancreatic tumors. Cancers. (2012) 4:989–1035. 10.3390/cancers4040989 24213498 PMC3712732

[147] Sayan AE Griffiths TR Pal R Browne GJ Ruddick A Yagci T. SIP1 protein protects cells from DNA damage-induced apoptosis and has independent prognostic value in bladder cancer. Proc Natl Acad Sci USA. (2009) 106:14884–9. 10.1073/pnas.0902042106 19706487 PMC2736415

[148] Suresh R Ali S Ahmad A Philip PA Sarkar FH. The role of cancer stem cells in recurrent and drug-resistant lung cancer. Adv Exp Med Biol. (2016) 890:57–74. 10.1007/978-3-319-24932-2_4 26703799

[149] Frank NY Schatton T Frank MH. The therapeutic promise of the cancer stem cell concept. J Clin Invest. (2010) 120:41–50. 10.1172/JCI41004 20051635 PMC2798700

[150] Ramos EK Hoffmann AD Gerson SL Liu H. New opportunities and challenges to defeat cancer stem cells. Trends Cancer. (2017) 3:780–96. 10.1016/j.trecan.2017.08.007 29120754 PMC5958547

[151] Dragu DL Necula LG Bleotu C Diaconu CC Chivu-Economescu M. Therapies targeting cancer stem cells: current trends and future challenges. World J Stem Cells. (2015) 7:1185–201. 10.4252/wjsc.v7.i9.1185 26516409 PMC4620424

[152] Majeti R. Monoclonal antibody therapy directed against human acute myeloid leukemia stem cells. Oncogene. (2011) 30:1009–19. 10.1038/onc.2010.511 21076471

[153] Deonarain MP Kousparou CA Epenetos AA. Antibodies targeting cancer stem cells: a new paradigm in immunotherapy? MAbs. (2009) 1:12–25. 10.4161/mabs.1.1.7347 20046569 PMC2715180

[154] Song Y Kim IK Choi I Kim SH Seo HR. Oxytetracycline have the therapeutic efficiency in CD133(+) HCC population through suppression CD133 expression by decreasing of protein stability of CD133. Sci Rep. (2018) 8:16100. 10.1038/s41598-018-34301-1 30382122 PMC6208387

[155] Huang YF Niu WB Hu R Wang LJ Huang ZY Ni SH. FIBP knockdown attenuates growth and enhances chemotherapy in colorectal cancer via regulating GSK3beta-related pathways. Oncogenesis. (2018) 7:77. 10.1038/s41389-018-0088-9 30275459 PMC6167373

[156] Li L Yang J Wang J Kopecek J. Amplification of CD20 cross-linking in rituximab-resistant B-lymphoma cells enhances apoptosis induction by drug-free macromolecular therapeutics. ACS Nano. (2018) 12:3658–70. 10.1021/acsnano.8b00797 29595951 PMC5916500

[157] Setubal Destro Rodrigues MF Gammon L Rahman MM Biddle A Nunes FD Mackenzie IC. Effects of Cetuximab and Erlotinib on the behaviour of cancer stem cells in head and neck squamous cell carcinoma. Oncotarget. (2018) 9:13488–500. 10.18632/oncotarget.24416 29568372 PMC5862593

[158] Tanei T Choi DS Rodriguez AA Liang DH Dobrolecki L Ghosh M. Antitumor activity of Cetuximab in combination with Ixabepilone on triple negative breast cancer stem cells. Breast Cancer Res. (2016) 18:6. 10.1186/s13058-015-0662-4 26757880 PMC4711100

[159] Sato F Kubota Y Natsuizaka M Maehara O Hatanaka Y Marukawa K. EGFR inhibitors prevent induction of cancer stem-like cells in esophageal squamous cell carcinoma by suppressing epithelial-mesenchymal transition. Cancer Biol Ther. (2015) 16:933–40. 10.1080/15384047.2015.1040959 25897987 PMC4623069

[160] McIntosh K Balch C Tiwari AK. Tackling multidrug resistance mediated by efflux transporters in tumor-initiating cells. Expert Opin Drug Metab Toxicol. (2016) 12:633–44. 10.1080/17425255.2016.1179280 27116192

[161] Deng J Shao J Markowitz JS An G. ABC transporters in multi-drug resistance and ADME-Tox of small molecule tyrosine kinase inhibitors. Pharm Res. (2014) 31:2237–55. 10.1007/s11095-014-1389-0 24842659

[162] Garg M. Targeting microRNAs in epithelial-to-mesenchymal transition-induced cancer stem cells: therapeutic approaches in cancer. Expert Opin Ther Targets. (2015) 19:285–97. 10.1517/14728222.2014.975794 25563894

[163] Yu F Deng H Yao H Liu Q Su F Song E. Mir-30 reduction maintains self-renewal and inhibits apoptosis in breast tumor-initiating cells. Oncogene. (2010) 29:4194–204. 10.1038/onc.2010.167 20498642

[164] Wang Y He L Du Y Zhu P Huang G Luo J. The long noncoding RNA lncTCF7 promotes self-renewal of human liver cancer stem cells through activation of Wnt signaling. Cell Stem Cell. (2015) 16:413–25. 10.1016/j.stem.2015.03.003 25842979

[165] Chakraborty C Sharma AR Sharma G Doss CGP Lee SS. Therapeutic miRNA and siRNA: moving from bench to clinic as next generation medicine. Mol Ther Nucleic Acids. (2017) 8:132–43. 10.1016/j.omtn.2017.06.005 28918016 PMC5496203

[166] van Zandwijk N Pavlakis N Kao SC Linton A Boyer MJ Clarke S. Safety and activity of microRNA-loaded minicells in patients with recurrent malignant pleural mesothelioma: a first-in-man, phase 1, open-label, dose-escalation study. Lancet Oncol. (2017) 18:1386–96. 10.1016/S1470-2045(17)30621-6 28870611

[167] Ashkenazi A. Targeting the extrinsic apoptosis pathway in cancer. Cytokine Growth Factor Rev. (2008) 19:325–31. 10.1016/j.cytogfr.2008.04.001 18495520

[168] Recio-Boiles A Ilmer M Rhea PR Kettlun C Heinemann ML Ruetering J. JNK pathway inhibition selectively primes pancreatic cancer stem cells to TRAIL-induced apoptosis without affecting the physiology of normal tissue resident stem cells. Oncotarget. (2016) 7:9890–906. 10.18632/oncotarget.7066 26840266 PMC4891091

[169] Guzman ML Swiderski CF Howard DS Grimes BA Rossi RM Szilvassy SJ. Preferential induction of apoptosis for primary human leukemic stem cells. Proc Natl Acad Sci USA. (2002) 99:16220–5. 10.1073/pnas.252462599 12451177 PMC138592

[170] Matsushita H Scaglioni PP Bhaumik M Rego EM Cai LF Majid SM. *In vivo* analysis of the role of aberrant histone deacetylase recruitment and RAR alpha blockade in the pathogenesis of acute promyelocytic leukemia. J Exp Med. (2006) 203:821–8. 10.1084/jem.20050616 16549595 PMC2118271

[171] Campos B Wan F Farhadi M Ernst A Zeppernick F Tagscherer KE. Differentiation therapy exerts antitumor effects on stem-like glioma cells. Clin Cancer Res. (2010) 16:2715–28. 10.1158/1078-0432.CCR-09-1800 20442299

[172] Ginestier C Wicinski J Cervera N Monville F Finetti P Bertucci F. Retinoid signaling regulates breast cancer stem cell differentiation. Cell Cycle. (2009) 8:3297–302. 10.4161/cc.8.20.9761 19806016 PMC2861502

[173] Relation T Dominici M Horwitz EM. Concise review: an (Im)Penetrable shield: how the tumor microenvironment protects cancer stem cells. Stem Cells. (2017) 35:1123–30. 10.1002/stem.2596 28207184

[174] Valkenburg KC de Groot AE Pienta KJ. Targeting the tumour stroma to improve cancer therapy. Nat Rev Clin Oncol. (2018) 15:366–81. 10.1038/s41571-018-0007-1 29651130 PMC5960434

[175] Nilendu P Sarode SC Jahagirdar D Tandon I Patil S Sarode GS. Mutual concessions and compromises between stromal cells and cancer cells: driving tumor development and drug resistance. Cell Oncol. (2018) 41:353–67. 10.1007/s13402-018-0388-2 30027403 PMC12995248

[176] Warner KA Miyazawa M Cordeiro MM Love WJ Pinsky MS Neiva KG. Endothelial cells enhance tumor cell invasion through a crosstalk mediated by CXC chemokine signaling. Neoplasia. (2008) 10:131–9. 10.1593/neo.07815 18283335 PMC2244688

[177] Zhang Z Dong Z Lauxen IS Filho MS Nor JE. Endothelial cell-secreted EGF induces epithelial to mesenchymal transition and endows head and neck cancer cells with stem-like phenotype. Cancer Res. (2014) 74:2869–81. 10.1158/0008-5472.CAN-13-2032 24686166 PMC4028029

[178] Gu JW Rizzo P Pannuti A Golde T Osborne B Miele L. Notch signals in the endothelium and cancer “stem-like” cells: opportunities for cancer therapy. Vasc Cell. (2012) 4:7. 10.1186/2045-824X-4-7 22487493 PMC3348040

[179] Barone A Sengupta R Warrington NM Smith E Wen PY Brekken RA. Combined VEGF and CXCR4 antagonism targets the GBM stem cell population and synergistically improves survival in an intracranial mouse model of glioblastoma. Oncotarget. (2014) 5:9811–22. 10.18632/oncotarget.2443 25238146 PMC4259439

[180] Hamerlik P Lathia JD Rasmussen R Wu Q Bartkova J Lee M. Autocrine VEGF-VEGFR2-Neuropilin-1 signaling promotes glioma stem-like cell viability and tumor growth. J Exp Med. (2012) 209:507–20. 10.1084/jem.20111424 22393126 PMC3302235

[181] Zhao J Ma MZ Ren H Liu Z Edelman MJ Pan H. Anti-HDGF targets cancer and cancer stromal stem cells resistant to chemotherapy. Clin Cancer Res. (2013) 19:3567–76. 10.1158/1078-0432.CCR-12-3478 23695169 PMC3707124

[182] Conley SJ Gheordunescu E Kakarala P Newman B Korkaya H Heath AN. Antiangiogenic agents increase breast cancer stem cells via the generation of tumor hypoxia. Proc Natl Acad Sci USA. (2012) 109:2784–9. 10.1073/pnas.1018866109 22308314 PMC3286974

[183] Rankin EB Nam JM Giaccia AJ. Hypoxia: signaling the metastatic cascade. Trends Cancer. (2016) 2:295–304. 10.1016/j.trecan.2016.05.006 28741527 PMC5808868

[184] Zhen Z Tang W Wang M Zhou S Wang H Wu Z. Protein nanocage mediated fibroblast-activation protein targeted photoimmunotherapy to enhance cytotoxic t cell infiltration and tumor control. Nano Lett. (2017) 17:862–9. 10.1021/acs.nanolett.6b04150 28027646

[185] Hernandez JL Padilla L Dakhel S Coll T Hervas R Adan J. Therapeutic targeting of tumor growth and angiogenesis with a novel anti-S100A4 monoclonal antibody. PLoS ONE. (2013) 8:e72480. 10.1371/journal.pone.0072480 24023743 PMC3762817

[186] Szot C Saha S Zhang XM Zhu Z Hilton MB Morris K. Tumor stroma-targeted antibody-drug conjugate triggers localized anticancer drug release. J Clin Invest. (2018) 128:2927–43. 10.1172/JCI120481 29863500 PMC6025988

[187] Najafi M Farhood B Mortezaee K. Cancer stem cells (CSCs) in cancer progression and therapy. J Cell Physiol. (2019) 234:8381–95. 10.1002/jcp.27740 30417375

[188] Zuo ZQ Chen KG Yu XY Zhao G Shen S Cao ZT. Promoting tumor penetration of nanoparticles for cancer stem cell therapy by TGF-beta signaling pathway inhibition. Biomaterials. (2016) 82:48–59. 10.1016/j.biomaterials.2015.12.014 26751819

[189] Yu B Wu K Wang X Zhang J Wang L Jiang Y. Periostin secreted by cancer-associated fibroblasts promotes cancer stemness in head and neck cancer by activating protein tyrosine kinase 7. Cell Death Dis. (2018) 9:1082. 10.1038/s41419-018-1116-6 30348980 PMC6197282

[190] Zhao ZK Yu HF Wang DR Dong P Chen L Wu WG. Overexpression of lysine specific demethylase 1 predicts worse prognosis in primary hepatocellular carcinoma patients. World J Gastroenterol. (2012) 18:6651–6. 10.3748/wjg.v18.i45.6651 23236241 PMC3516205

[191] Su S Chen J Yao H Liu J Yu S Lao L. CD10(+)GPR77(+) cancer-associated fibroblasts promote cancer formation and chemoresistance by sustaining cancer stemness. Cell. (2018) 172:841–56.e16. 10.1016/j.cell.2018.01.009 29395328

[192] Bonde AK Tischler V Kumar S Soltermann A Schwendener RA. Intratumoral macrophages contribute to epithelial-mesenchymal transition in solid tumors. BMC Cancer. (2012) 12:35. 10.1186/1471-2407-12-35 22273460 PMC3314544

[193] Su S Liu Q Chen J Chen J Chen F He C. A positive feedback loop between mesenchymal-like cancer cells and macrophages is essential to breast cancer metastasis. Cancer Cell. (2014) 25:605–20. 10.1016/j.ccr.2014.03.021 24823638

[194] Valeta-Magara A Gadi A Volta V Walters B Arju R Giashuddin S Inflammatory breast cancer promotes development of M2 tumor-associated macrophages and cancer mesenchymal cells through a complex cytokine network. Cancer Res. (2019) 79:3360–71. 10.1158/0008-5472.CAN-17-2158 31043378 PMC7331114

[195] Su YJ Lin WH Chang YW Wei KC Liang CL Chen SC. Polarized cell migration induces cancer type-specific CD133/integrin/Src/Akt/GSK3beta/beta-catenin signaling required for maintenance of cancer stem cell properties. Oncotarget. (2015) 6:38029–45. 10.18632/oncotarget.5703 26515729 PMC4741982

[196] Huang WC Kuo KT Wang CH Yeh CT Wang Y. Cisplatin resistant lung cancer cells promoted M2 polarization of tumor-associated macrophages via the Src/CD155/MIF functional pathway. J Exp Clin Cancer Res. (2019) 38:180. 10.1186/s13046-019-1166-3 31036057 PMC6489343

[197] Li X Bu W Meng L Liu X Wang S Jiang L. CXCL12/CXCR4 pathway orchestrates CSC-like properties by CAF recruited tumor associated macrophage in OSCC. Exp Cell Res. (2019) 378:131–8. 10.1016/j.yexcr.2019.03.013 30857971

[198] Majeti R Chao MP Alizadeh AA Pang WW Jaiswal S Gibbs KD Jr. CD47 is an adverse prognostic factor and therapeutic antibody target on human acute myeloid leukemia stem cells. Cell. (2009) 138:286–99. 10.1016/j.cell.2009.05.045 19632179 PMC2726837

[199] Chao MP Alizadeh AA Tang C Jan M Weissman-Tsukamoto R Zhao F. Therapeutic antibody targeting of CD47 eliminates human acute lymphoblastic leukemia. Cancer Res. (2011) 71:1374–84. 10.1158/0008-5472.CAN-10-2238 21177380 PMC3041855

[200] Chao MP Alizadeh AA Tang C Myklebust JH Varghese B Gill S. Anti-CD47 antibody synergizes with rituximab to promote phagocytosis and eradicate non-Hodgkin lymphoma. Cell. (2010) 142:699–713. 10.1016/j.cell.2010.07.044 20813259 PMC2943345

[201] Advani R Flinn I Popplewell L Forero A Bartlett NL Ghosh N. CD47 Blockade by Hu5F9-G4 and Rituximab in Non-Hodgkin's Lymphoma. N Engl J Med. (2018) 379:1711–21. 10.1056/NEJMoa1807315 30380386 PMC8058634

[202] Logtenberg MEW Jansen JHM Raaben M Toebes M Franke K Brandsma AM. Glutaminyl cyclase is an enzymatic modifier of the CD47- SIRPalpha axis and a target for cancer immunotherapy. Nat Med. (2019) 25:612–9. 10.1038/s41591-019-0356-z 30833751 PMC7025889

[203] Wang Y Wang B Xiao S Li Y Chen Q. miR-125a/b inhibits tumor-associated macrophages mediated in cancer stem cells of hepatocellular carcinoma by targeting CD90. J Cell Biochem. (2019) 120:3046–55. 10.1002/jcb.27436 30536969

[204] Fan Z Cui H Yu H Ji Q Kang L Han B MiR-125a promotes paclitaxel sensitivity in cervical cancer through altering STAT3 expression. Oncogenesis. (2016) 5:e197 10.1038/oncsis.2016.1 26878391 PMC5154343

[205] Tang L Shen H Li X Li Z Liu Z Xu J. MiR-125a-5p decreases after long non-coding RNA HOTAIR knockdown to promote cancer cell apoptosis by releasing caspase 2. Cell Death Dis. (2016) 7:e2137. 10.1038/cddis.2016.41 26962687 PMC4823942

[206] Hu J Zheng L Shen X Zhang Y Li C Xi T. MicroRNA-125b inhibits AML cells differentiation by directly targeting Fes. Gene. (2017) 620:1–9. 10.1016/j.gene.2017.04.002 28389358

[207] Delyon J Mateus C Lefeuvre D Lanoy E Zitvogel L Chaput N. Experience in daily practice with ipilimumab for the treatment of patients with metastatic melanoma: an early increase in lymphocyte and eosinophil counts is associated with improved survival. Ann Oncol. (2013) 24:1697–703. 10.1093/annonc/mdt027 23439861

[208] Topalian SL Hodi FS Brahmer JR Gettinger SN Smith DC McDermott DF. Safety, activity, and immune correlates of anti-PD-1 antibody in cancer. N Engl J Med. (2012) 366:2443–54. 10.1056/NEJMoa1200690 22658127 PMC3544539

[209] Phung CD Nguyen HT Tran TH Choi HG Yong CS Kim JO. Rational combination immunotherapeutic approaches for effective cancer treatment. J Control Release. (2019) 294:114–30. 10.1016/j.jconrel.2018.12.020 30553850

[210] Ruan H Bu L Hu Q Cheng H Lu W Gu Z. Strategies of combination drug delivery for immune checkpoint blockades. Adv Healthc Mater. (2019) 8:e1801099. 10.1002/adhm.201801099 30548835

[211] Shi X Zhang X Li J Mo L Zhao H Zhu Y. PD-1 blockade enhances the antitumor efficacy of GM-CSF surface-modified bladder cancer stem cells vaccine. Int J Cancer. (2018) 142:2106–17. 10.1002/ijc.31219 29243219

[212] Zheng F Dang J Zhang H Xu F Ba D Zhang B. Cancer stem cell vaccination with PD-L1 and CTLA-4 blockades enhances the eradication of melanoma stem cells in a mouse tumor model. J Immunother. (2018) 41:361–8. 10.1097/CJI.0000000000000242 30063587 PMC6128768

[213] Brentjens RJ Davila ML Riviere I Park J Wang X Cowell LG. CD19-targeted T cells rapidly induce molecular remissions in adults with chemotherapy-refractory acute lymphoblastic leukemia. Sci Transl Med. (2013) 5:177ra38. 10.1126/scitranslmed.3005930 23515080 PMC3742551

[214] Maus MV. Designing CAR T cells for glioblastoma. Oncoimmunology. (2015) 4:e1048956. 10.1080/2162402X.2015.1048956 26587317 PMC4635938

[215] Deng Z Wu Y Ma W Zhang S Zhang YQ. Adoptive T-cell therapy of prostate cancer targeting the cancer stem cell antigen EpCAM. BMC Immunol. (2015) 16:1. 10.1186/s12865-014-0064-x 25636521 PMC4318439

[216] Li H Huang Y Jiang DQ Cui LZ He Z Wang C. Antitumor activity of EGFR-specific CAR T cells against non-small-cell lung cancer cells *in vitro* and in mice. Cell Death Dis. (2018) 9:177. 10.1038/s41419-017-0238-6 29415996 PMC5833445

